# Penicillin Allergy Testing and Delabeling for Patients Who Are Prescribed Penicillin: A Systematic Review for a World Health Organization Guideline

**DOI:** 10.1007/s12016-024-08988-2

**Published:** 2024-05-02

**Authors:** Rui Providencia, Ghazaleh Aali, Fang Zhu, Brian F. Leas, Rachel Orrell, Mahmood Ahmad, Jonathan J. H. Bray, Ferruccio Pelone, Petra Nass, Eloi Marijon, Miryan Cassandra, David S. Celermajer, Farhad Shokraneh

**Affiliations:** 1 University College London, London, UK; 2 Barts Heart Centre, London, UK; 3 Department of Evidence Synthesis, Systematic Review Consultants LTD, Oxford, UK; 4 Department of Biostatistics, Systematic Review Consultants LTD, Oxford, UK; 5 Royal Free London NHS Foundation Trust, London, UK; 6 European Georges Pompidou Hospital, Paris, France; 7 Hospital Dr. Ayres de Menezes, São Tomé, São Tomé and Príncipe; 8 The University of Sydney, Sydney, New South Wales, Australia

**Keywords:** Rheumatic fever, Rheumatic heart disease, Penicillin, Allergy

## Abstract

Secondary prevention with penicillin aims to prevent further episodes of acute rheumatic fever and subsequent development of rheumatic heart disease (RHD). Penicillin allergy, self-reported by 10% of the population, can affect secondary prevention programs. We aimed to assess the role for (i) routine penicillin allergy testing and the (ii) safety of penicillin allergy delabeling approaches in this context. We searched MEDLINE, Embase, CENTRAL, ClinicalTrials.gov, WHO ICTRP, ISRCTN, and CPCI-S to identify the relevant reports. We found 2419 records, but no studies addressed our initial question. Following advice from the WHO-Guideline committee and experts, we identified 6 manuscripts on allergy testing focusing on other populations showing that the prevalence of allergy confirmed by testing was low and the incidence of life-threatening reactions to BPG was very low (< 1–3/1000 individuals treated). A subsequent search addressed penicillin allergy delabeling. This found 516 records, and 5 studies addressing the safety of direct oral drug challenge vs. skin testing followed by drug administration in patients with suspected penicillin allergy. Immediate allergic reactions of minor severity were observed for a minority of patients and occurred less frequently in the direct drug challenge group: 2.3% vs. 11.5%; RR = 0.25, 95%CI 0.15–0.45, *P* < 0.00001, *I*^2^ = 0%. No anaphylaxis or deaths were observed. Severe allergic reactions to penicillin are extremely rare and can be recognized and dealt by trained healthcare workers. Confirmation of penicillin allergy diagnosis or delabeling using direct oral drug challenge or penicillin skin testing seems to be safe and is associated with a low rate of adverse reactions.

**Supplementary Information:**

The online version contains supplementary material available at 10.1007/s12016-024-08988-2

## Background

Acute Rheumatic Fever (ARF) is an inflammatory condition that may occur 10 to 21 days after an upper respiratory tract infection caused by group A beta‐haemolytic streptococci strains [1, 2]. Secondary prevention with antibiotics aims to prevent further episodes of ARF and subsequent development of rheumatic heart disease (RHD) [3].

A report by the World Health Organization (WHO) in 2018 utilizing data from the Global Burden of Disease Study 2016 revealed that 30 million people were thought to be affected by RHD globally at that time and that in 2015 RHD had caused 305,000 deaths and 11.5 million disability-adjusted life years lost [4]. The most affected regions, accounting for 84% of all prevalent cases and 80% of all estimated deaths, were Africa, South-East Asia, and the Western Pacific regions. India, China, Egypt, Sudan, and Yemen, and particular areas of Australia, New Zealand, and the Pacific island States, seem to be high-prevalence territories of concern [4, 5]. However, regional burdens may have been underestimated due to lack of good or reliable data in some areas of the World. 

Potential issues may arise in patients treated with BPG, namely a penicillin allergy label. The fraction of the population around the world labeled with a penicillin allergy varies widely and appears related to antibiotic usage patterns [6, 7]. Only about 2% of individuals who used healthcare in Hong Kong carried a penicillin allergy label [8]. Approximately 4% of individuals who sought medical care in Colombia carried a penicillin allergy label [9]. Regional figures for penicillin allergy labeled hospitalized patients from mainland China and Japan ranged from 5 to 5.6% [10–12]. Up to 6% of a population-based cohort in the UK carried a penicillin allergy label [13]. Just under 10% of individuals who used healthcare in the USA carried a penicillin allergy label [14]. About 12% of a sample of children and adults being seen as outpatients in Belgium carried a penicillin allergy label [15]. Overall, the prevalence of a penicillin allergy label was higher in individuals who actively used healthcare, in females, in hospitalized patients, and increased with increasing age [16, 17]. The prevalence of penicillin allergy in much of the world remains unknown. Less than 5% of individuals labeled with a penicillin allergy are confirmed, with appropriate testing, to have either a currently active acute onset IgE-mediated penicillin allergy or a clinically significant delayed onset T-cell-mediated penicillin hypersensitivity [18].

Allergic reactions are amplified reactions due an immune mechanism to an otherwise harmless and exogenous compound. Depending on the timing following contact with the compound, these can be immediate (< 1 h: urticaria, angioedema, and anaphylaxis), accelerated (1 to 72 h: urticaria and maculopapular rashes), or late (> 72 h: skin rashes, erythema multiforme, serum sickness, and hemolytic anemia) [19]. Although rare, allergic reactions can be severe (e.g., angioedema and anaphylaxis, Stevens-Johnson syndrome, toxic epidermal necrolysis).

Anaphylaxis and death following penicillin administration is of special concern in RHD with anecdotal cases reported across the globe motivating concerns in patients and health professionals, and causing devastating effect on RHD program, from discontinuation of benzathine penicillin G (BPG) prophylaxis to banning of BPG in some countries [20]. The mechanism for anaphylaxis is IgE-mediated. Risk factors associated with IgE-mediated reactions include age, presence of allergic diseases, and multiple short courses (mainly if parenteral or topic) [19].

Testing for penicillin allergy could be an approach to optimize antibiotic selection and improve patient safety by preventing allergic reactions. In the general population, the current practices vary and are based on testing patients when they are having their first injection, asking the patients about their previous history of allergic reactions or looking at their medical records, and administration of the penicillin injection in a healthcare facility equipped to face possible anaphylaxis or severe reactions [21]. Despite being done in some areas of the World like Chinese mainland, where due to regulation an intradermal test is routinely performed before using penicillins [22], or frequently before BPG administration for ARF prevention, according to 40% of respondents of an African health worker survey [23], routine penicillin allergy testing in individuals without reported allergy and requiring therapy with a penicillin is not usually recommended due to the very low rate of anaphylaxis [24].

People who self-report as penicillin allergic based on unspecific reactions in the past, and in the absence of confirmatory testing, frequently do not have a true penicillin allergy. Removing incorrect penicillin allergy labels (i.e., penicillin allergy delabeling) is of importance to improve antimicrobial stewardship practices worldwide [19]. Delabeling can be done through oral administration of a low-dose penicillin in low-risk penicillin allergy patients, and, on some occasions, may be done directly by clinical history taking alone [25]. The latter approach can also be followed in e-consults [26], obliviating the need for any further workup, namely when the reported reactions are clearly nonimmunologic (e.g., nausea, headache, or fatigue).

We aimed to assess the role for (i) routine penicillin allergy testing and the (ii) safety of penicillin allergy delabeling approaches in individuals prescribed with penicillin or with a suspected or reported penicillin allergy.

## Methods

### Search Strategy/Key Words/Databases

On 2nd October 2022, we searched the following sources from the inception up to the search date:

- Cochrane Central Register of Controlled Trials = CENTRAL (the last available issue)
- ClinicalTrials.gov (up to present)
- Conference Proceedings Citation Index-Science = CPCI-S (1990–present)
- Embase via Ovid SP (1974–present)
- ISRCTN.com (up to present)
- MEDLINE via Ovid SP (1946–present)
- WHO ICTRP (up to present)

Search strategies were developed by consulting the clinicians, controlled vocabularies (Medical Subject Headings = MeSH and Excerpta Medica Tree = Emtree), literature review, and test search results. Based on the recommendations from the 2nd edition of the Cochrane Handbook for Systematic Reviews of Interventions [27], the searches were balanced between the sensitivity and specificity of the search results without applying a methodological search filter. Furthermore, the search was not limited to publication date, publication language, publication status, or document type.

The search strategies were peer-reviewed by another Information Specialist before the final run. The searches were run, documented, and reported by a senior information scientist and followed the globally accepted guidelines: PRISMA 2020 [28], PRISMA-S [29], and PRESS [30]. Search strategies are available in Appendix 1.

We contacted the authors of the studies as-required to obtain the data or information.

### Inclusion/Exclusion Criteria

#### Population

Patients prescribed IM BPG for secondary prevention of RHD.

#### Intervention

Different types of allergy tests for penicillin can be used in individuals with a reported or suspected penicillin allergy [20]:

- Laboratory or in vitro diagnostics (measuring tryptase or specific IgE antibodies, and cellular in vitro testing)
- Penicillin skin testing (PST) (patch test, the skin prick test, and the intradermal test) can detect the presence/absence of specific anti-penicillin IgE antibodies against minor (benzylpenicillin, benzylpenicilloate, and benzylpenilloate) or major determinants (penicilloyl-polylysine). During this test, the healthcare worker administers the test solution to the skin with a tiny needle. A positive reaction presents as redness, itchiness, and a raised bump. A positive result indicates a high likelihood of penicillin allergy. However, due to the high rate of positive tests of routine testing, which can be as high as 5% [31], the possibility of a false positive penicillin skin test also has to be considered. False-positives can be due to test (e.g., 3-mm wheal threshold, higher concentrations of reagents, or improper preparation or storage) or patient factors (e.g., female sex) [24].
- Drug challenge (DC), or graded challenge, or test dosing, can be performed following a negative lab or skin test and will exclude or confirm a penicillin allergy diagnosis. This consists of the administration of penicillin under strict clinical supervision, starting with a very low dose, and subsequently administering more drug [32].

#### Comparator

No allergy test for penicillin.

#### Types of Studies

We planned to include studies with a patient control group:

- Cohort studies
- Cross-sectional studies with a control group
- Case–control studies
- Randomized controlled trials (RCTs)
- Controlled clinical trials
- Non-randomized clinical trials

We excluded a study if the only available control groups were healthy people and the single-arm self-control study when the same patients acted as their own control. If a study used a cross-over design, we only used the data before the cross-over stage, if available.

We did not expect to find cluster RCTs; however, if found, we planned to follow Cochrane’s methods dealing with the risk of bias assessment and meta-analysis [33].

#### Primary Outcome

- Allergic reactions to penicillin

#### Secondary Outcomes

- Anaphylactic shocks
- Vasovagal reactions
- Arrhythmia rate as defined and reported based on the study protocol
- Treatment adherence: We will report this outcome as a percentage of those who received 100% of their prescribed BPG or as a percentage of the total number of recommended injections administered relative to the study population
- Acceptability to provider and patient as defined in the study protocol
- Adverse events (any)
- Serious adverse events (any)

### How Studies Were Selected Based on Titles and Abstracts/Full Papers

The search results were imported into EndNote 20, and the duplicates were removed. The remaining records were then imported into Rayyan for double-blind screening by two reviewers. The blinding was inactivated when the screening was finished to resolve the conflicts.

### Data Extraction

We used Microsoft Excel and Review Manager 5.4 (RevMan) for data management and analysis [34].

A reviewer extracted the data, and another independently double-checked the extracted and analyzed data from all studies.

### Quality Assessment

#### Risk of Bias Assessment

For randomized controlled trials, we planned to use the default data extraction and risk of bias assessment tool embedded within RevMan. The first version of the Risk of Bias tool [35] was used due to concerns regarding the difficulty of version 2, even for experienced users, which may result in delays in publication [36]. This tool consists of seven domains and allows three levels of judgements (low, unclear, or high):

- Random sequence generation (selection bias)
- Allocation concealment (selection bias)
- Blinding of participants and personnel (performance bias)
- Blinding of outcome assessment (detection bias)
- Incomplete outcome data (attrition bias)
- Selective reporting (reporting bias)
- Other biases

For non-randomized controlled studies, we planned to use the Effective Public Health Practice Project Quality Assessment Tool (EPHPP) [37], which assesses eight categories of bias and assigns three levels of strong, moderate, and weak to each of these bias areas:

- Selection bias
- Study design
- Confounders
- Blinding
- Data collection methods
- Withdrawals and drop-outs
- Intervention integrity
- Analyses

#### Assessment of Reporting Biases

We planned that if this review includes 10 or more studies, we create and examine a funnel plot to explore possible small‐study biases for the primary outcomes. We also planned to perform a formal statistical test for asymmetry in this case. Since there was only a small number of included studies, the ability to detect publication bias was diminished, so other sources of asymmetry, such as other dissemination biases, differences in the quality of smaller studies, the existence of true heterogeneity, and chance, were considered [38].

### GRADE Methodology

We planned to use the five GRADE considerations (study limitations, consistency of effect, imprecision, indirectness, and publication bias) to assess the certainty of the body of evidence as it relates to the studies which contribute data to the meta‐analyses for the prespecified outcomes. We planned to use methods and recommendations described in the Cochrane Handbook for Systematic Reviews of Interventions [27] using GRADEpro GDT software [39]. The overall “Risk of bias” judgement for each study was planned to be used as part of the GRADE assessment of study limitations. The first version of the Risk of Bias tool [35] was planned to be used due to concerns regarding the difficulty of version 2, even for experienced users, which may result in delays in publication [36].

We justified all decisions to downgrade the evidence’s quality using footnotes and added comments to aid the reader’s understanding of the review if necessary.

### Data Synthesis Incl. Meta-analyses

For binary (dichotomous) outcomes, we planned to use Risk Ratios (RR). All measures were planned to be presented with a 95% confidence interval. We planned to enter data presented as a scale with a consistent direction of effect.

We planned to use a random-effects model due to the high probability of heterogeneity in the RCTs and other evidence that might have been included in this review.

We planned to use forest plots to visualize the meta-analysis results and the GRADE methodology for presenting the certainty of the evidence as explained by the Cochrane Handbook [40]. We planned to upload the RevMan files to GRADE GDT to create a summary of findings table for interventional studies.

We planned for all studies to be included in the primary analysis, and to assess the potential effects of studies at high risk or high risk/some concerns, we planned to carry out sensitivity analyses.

#### Assessment of Heterogeneity

We planned to inspect forest plots visually to consider the direction and magnitude of effects and the degree of overlap between confidence intervals. We planned to use the *I*^2^ and Tau^2^ statistics to measure heterogeneity among the studies in each analysis. We acknowledged that there is substantial uncertainty in the value of *I*^2^ when only a small number of studies exist. We also considered the *P*-value from the Chi^2^ test.

#### Subgroup Analysis

We planned to carry out subgroup analyses for the following factors for all outcomes but only for our primary time point of interest:

• Children

• Adolescents

• Pregnant women

• Other adults

We planned to use the formal test for subgroup differences in Review Manager 5.4 [34] and base our interpretation on this.

#### Changes from the Protocol

We identified no studies meeting our inclusion criteria. After this finding was presented to the WHO Guideline Committee in March 2023, we were advised to summarize the most relevant systematic reviews on this topic focusing on broader populations, with no limitation to healthcare conditions, to potentially inform the guideline development. This change was based on the assumption that evidence was required to address this question and allergic reactions are immunologically mediated and, there is no data suggesting a different behavior (incidence or severity) in RHD patients. We used the following simple search strategy in PubMed to identify the most relevant systematic reviews on 1st December 2022:

Penicillin*[TI] AND Allerg*[TI] AND Test*[TI] AND (Systematic Review*[TI] OR Meta-Analys*[TI])

During the WHO Guideline Committee in April 2023 on presenting these findings, there was a discussion on the prevalence of penicillin allergy labels, and how to approach these patients in areas of high prevalence of ARF/RHD when considered for penicillin treatment (or an alternative antibiotic if found to be truly penicillin allergic), as allergy testing may not be routinely available [41]. According to WHO guidance, this subgroup of patients (i.e., patients requiring penicillin of other antibiotics and labeled as allergic) should be carefully examined and their antibiotic risk level should be determined [42], hence they cannot be assessed using the original PICO which included “doing nothing” as the comparator. Discussion in the April meeting covered the role of PST, and potential alternatives, as low-dose direct DC, for confirming or removing a penicillin allergy label. To address this specific sub-group that would not be addressed by the initial PICO question, we ran a simple search strategy on the Cochrane Central Register of Controlled Trials (Issue 7 of 12, July 2023), PubMed, and Embase on 31st of July 2023 using the expression: “penicillin allergy” AND “trial.” The aim was to understand in patients with suspected penicillin allergy what is the safest method to delabel a penicillin allergy or to confirm a diagnosis. Studies were identified using the same inclusion and exclusion criteria, but the PICO had changes to the population and comparator:

- Population: patients with suspected penicillin allergy (self-reported by the patient or their guardian, or patient history);
- Intervention: as before (laboratory investigations, PST, or direct DC).
- Comparator: different approach to the one used in the intervention group.
- Outcomes: as before (primary endpoint: allergic reactions to penicillin; secondary endpoints: anaphylactic shocks, vasovagal reactions, arrhythmias, treatment adherence, acceptability and adverse events).

## Results

### Study Selection

#### Routine Penicillin Allergy Testing for Patients Prescribed Penicillin for Secondary Prevention of RHD

**Fig. 1:**
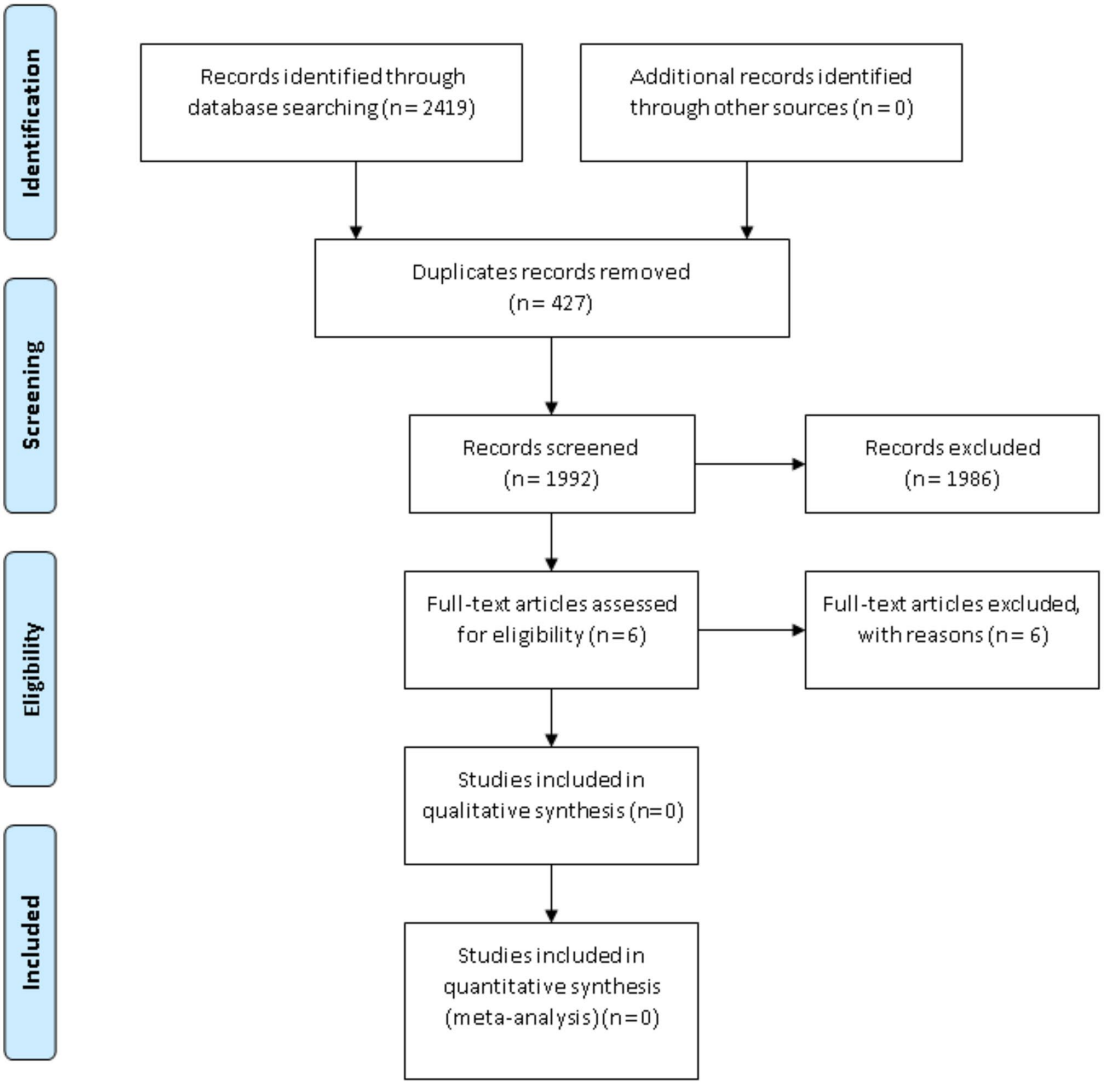
PRISMA flow chart illustrating screening and study selection for the first PICO and question—“Should all patients who are prescribed intramuscular BPG for secondary prevention of RHD be tested for penicillin allergy and if so, which is the best method?”

After assessing 2419 records and obtaining the full text for 6 reports, this review found no studies meeting the inclusion criteria (Fig. 1). We excluded five reports because they were cross-sectional prevalence studies with no control groups and one report for being a review (Supplementary material. Annex – Excluded studies). We assessed the review report for possible usable data or relevant references and did not find relevant information.

#### Systematic Reviews and Studies with Broader Population

An alternative approach to run a targeted search for relevant systematic reviews with broader population found four systematic reviews, and two additional studies were suggested by experts in the field.

A Bayesian meta-analysis by Cardoso-Fernandes and colleagues [32] looked at the frequency of severe reactions following penicillin DC in 112 primary studies including 26,595 participants with a penicillin allergy label who underwent DC. The pooled frequency of severe reactions was estimated at 0.06% (95% credible interval [95% CrI] 0.01–0.13%; *I*^2^ = 57.9%). Most severe reactions (80/93; 86.0%) consisted of anaphylaxis (estimated frequency: 0.03%, 95%CrI 0 to 0.04%; *I*^2^ = 44.2%). No patients had a subsequent fatal reaction reinforcing the safety of penicillin DC. As a limitation, the systematic review excluded studies assessing patients with specific diseases or occupations (indirectness).

Harandian and colleagues published a systematic review assessing the prevalence of immediate adverse reactions to penicillin derivatives, in patients with a reported adverse reaction to these antibiotics and the effect of age on the prevalence of reactions [43] . Their main inclusion criteria were PST or oral challenge in case of negative skin tests to establish immediate reactions. Fourteen studies were included (four only on children, six for adults, and four with mixed child and adult populations). Studies were from Denmark (*n* = 2), Italy (*n* = 2), Slovenia (*n* = 1), Spain (*n* = 1), Switzerland (*n* = 1), Thailand (*n* = 1), Turkey (*n* = 1), and the USA (*n* = 5). With a wide CI and high heterogeneity (*I*^2^ = 87.2 to 97.0%), a higher prevalence of allergic reactions was observed among adults than children. The prevalence of immediate reactions to penicillin derivatives in patients reporting a β-lactam hypersensitivity is 1.98% (95% CI, 1.35%, 2.60%) among children, 7.78% (95% CI, 6.53%, 9.04%) among adults, and 2.84% (95% CI, 1.77%, 3.91%) among mixed populations. As possible limitations, the review only included English and French literature published within a 5-year period (June 2010 to May 2015) and focused on immediate adverse reactions in patients with a reported adverse reaction.

Sacco and colleagues aimed to determine whether inpatient testing for penicillin allergy affects clinical outcomes during hospitalization [31]. They included any intervention to rule out penicillin allergy. Their systematic review included 24 studies (*N* = 24 to 252 participants), 18 using PST with or without oral amoxicillin challenge. Negative PST ranged from 79 to 100%. The population-weighted mean for a negative PST was 95.1% [CI 93.8–96.1]. Based on four studies, the testing was associated with decreased healthcare costs. Inpatient penicillin allergy testing result in: change in antibiotic selection—greater in ICU (77.97% [CI 72.0–83.1] versus 54.73% [CI 51.2–58.2], *P* < 0.01); increase in prescribing cephalosporin (range 10.7–48%) and penicillin (range 9.9–49%); decrease in using vancomycin and fluoroquinolone. The authors concluded the testing to be effective and safe to rule out the penicillin allergy and a negative test rate similar to perioperative and outpatient data. As limitations, the review focused on 15 years (until 6th December 2016) and only on English literature. Included studies had mixed design and quality and they included seven conference abstracts.

A case–control study of penicillin allergic members of Kaiser Permanent Southern California compared individuals who had penicillin allergy testing, in the setting of outpatient allergy consultation, vs matched controls who did not receive testing over a mean 3.6- to 4-year follow-up period [44]. Individuals who had penicillin allergy testing had higher exposure to penicillins and 1st and 2nd generation cephalosporins, alongside with fewer outpatient and emergency department visits, and fewer hospital days. The approach was safe, with no episodes of penicillin- or cephalosporin-associated serious cutaneous adverse reactions or anaphylaxis documented.

A systematic review by Sousa-Pinto and colleagues assessed the accuracy of penicillin allergy diagnostic tests (skin tests and specific IgE quantification) in the diagnostic evaluation of patients reporting a penicillin/beta-lactam allergy [45]. The review included 105 studies in patients reporting a penicillin allergy assessed with skin tests and/or specific IgE quantification using DC results as the reference. The accuracy of diagnostic tests was assessed with bivariate random-effects meta-analyses based on 27 studies from Canada (*n* = 3), Denmark (*n* = 1), Israel (*n* = 2), Italy (*n* = 5), Slovenia (*n* = 1), Spain (*n* = 5), Sweden (*n* = 1), Switzerland (*n* = 2), Thailand (*n* = 1), the UK (*n* = 2), and the USA (*n* = 4). Skin tests had summary sensitivity of 30.7% (95%CI 18.9–45.9%), specificity of 96.8% (95%CI 94.2–98.3%), and moderate discriminative capacity (c-statistic 0.686; 20 studies), and specific IgE quantification had summary sensitivity of 19.3% (95%CI 12.0–29.4%), specificity of 97.4% (95%CI 95.2–98.6%) and low discriminative capacity (c-statistic 0.420; 11 studies). Unfortunately, ARF was among the exclusion criteria in this review, posing some questions regarding indirectness.

During a period of shortage of PST reagents, between January 2007 and August 2009, Macy and colleagues prospectively evaluated 150 consecutive individuals with history of penicillin allergy using both commercial anti-penicillin IgE fluorometric enzyme immunoassays and PST [46]. The fluorometric enzyme immunoassays were negative for all 6 participants with positive PST and were also negative for the 3 participants with negative PST who subsequently had a positive oral challenge (one participant developed hives and two had non-urticarial rashes). Furthermore, all four participants with positive fluorometric enzyme assays had negative PST and negative oral challenge. These results provided evidence support to PST followed by oral challenge in case of negative PST, whilst showing that commercial fluorometric enzyme immunoassays were of no use for evaluating individuals with history of penicillin allergy.

#### Confirmation or Delabeling of Patients with Suspected Penicillin Allergy

##### Study Selection and Description

**Fig. 2:**
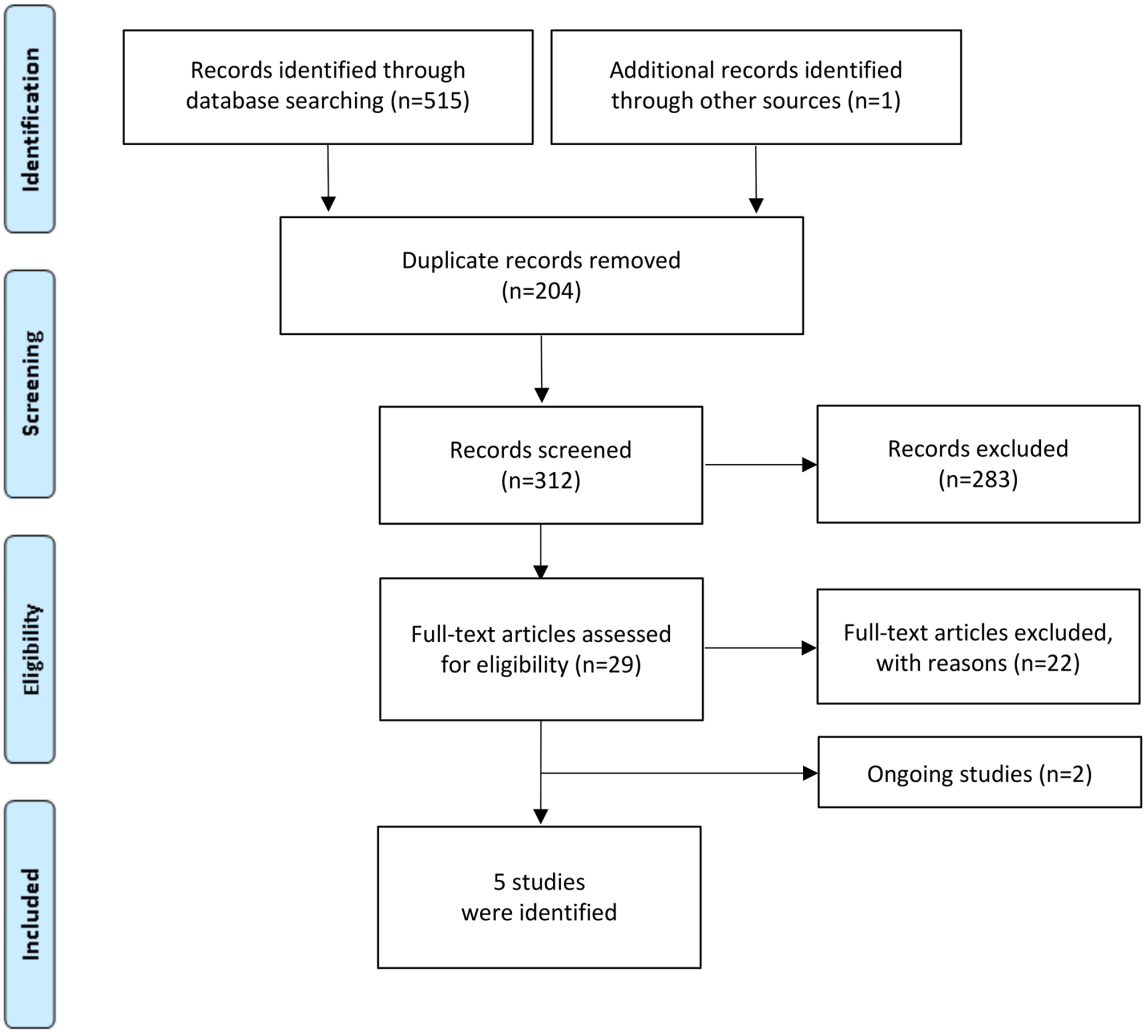
PRISMA flow chart illustrating screening and study selection for addressing penicillin allergy delabeling

After assessing 516 records and obtaining the full text for 29 reports, we found five studies meeting the inclusion criteria [47–51] (Fig. 2). We identified 2 additional ongoing trials who seem to meet the inclusion criteria [52, 53].

We excluded thirteen reports because they were single-arm studies with no control groups, two studies were systematic reviews, one study had the wrong population, two studies had the wrong comparator, one study had the wrong study design, and one was a health economics analysis.

Three of the included studies were randomized controlled trials [48, 50, 51], one was a single-arm trial with historical controls [47, 54], and one was a case–control study [49]. One study [51] was a conference abstract and we contacted the authors for further information on the study.

Included participants differed across studies, with two studies [49, 51] including only adults, and the remaining [47, 48, 50] included a mix of children and adults (Table 1). One study only included pregnant patients [51].

**Table 1:** Study design of included studies for the second PICO and question—“In patients with suspected penicillin allergy what is the best method to delabel a penicillin allergy or to confirm a diagnosis?”

**Study**	**Country**	**Study design**	**Number of patients**	**Study period**	**Setting**	**Patient selection (random/consecutive)**	**Patient type and exclusion criteria**
Iammatteo et al. (2019) [25]	USA	Single-arm trial with historical controls	321	DC: January 2016 to December 2017 PST: October 2010 to November 2015	Outpatient drug allergy clinic	DC: consecutive patients with reported penicillin allergy were screened for enrollment PST: retrospective chart review of all patients with a reported beta-lactam allergy	Aged ≥ 7 with historical non-life-threatening reactions to penicillin Excluded: pregnancy and antihistamine use within 3 days
Mustafa et al. (2019) [26]	USA	RCT	159	April to August 2018	Outpatient allergy practice	363 consecutive patients with reported penicillin allergy were approached and 185 (51%) completed the evaluation Reasons for deferral: - Patient time constraint—39 - Fear of needles/other—23 - Delabeled on history alone or family history only—25 - Provider time constraint—12 - Medication—16	Aged 5–17 with a history of cutaneous-only reaction to penicillin > 1 year ago, or aged ≥ 18 with a history of cutaneous-only reaction > 10 years ago Excluded: pregnancy, severe cutaneous non-IgE mediated reactions or serum-sickness-like reaction
Stevenson et al. (2019) [27]	Australia	Retrospective study	447	February 2016 to May 2018	Seven immunology outpatient clinics	All patients, aged ≥ 16 years who underwent PST and/or OPC for penicillin allergy assessment were included	Aged ≥ 16 years Some patients (*n* = 203) were classified as “high-risk”: index reaction < 1 year, or mucosal and/or systemic involvement Excluded: non-IgE mediated reactions, pregnancy, and significant cardiorespiratory comorbidities
Copaescu et al. (2023) (PALACE) [28]	USA, Canada, and Australia	RCT	382	June 2021 to December 2022	Six allergy outpatient clinics	Out of 446 consecutive patients labeled with a penicillin allergy referred to an allergy clinic meeting eligibility criteria, 382 were randomized Reasons for not being randomized: - Refusal of consent—21 - Reason not recorded—43	Aged ≥ 18 PEN-FAST score < 3 Excluded: drug-related anaphylaxis, chronic spontaneous urticaria or mast-cell disease, non-IgE-mediated severe reactions, and on antihistamine therapy
Ramsey et al. (2023) [29]	USA	RCT	38	N.A	N.A	N.A	Pregnant patients with a history of cutaneous-only or mild reactions to penicillin > 5 years ago Excluded: N.A

*RCT* randomized controlled trial, *N.A.* not available

Four studies included patients only with cutaneous or non-life-threatening reactions (47, 48, 50, 51). One study also included patients classified as high-risk (recent reactions/ < 1 year, and with mucosal or systemic involvement [49].

The direct DC was oral in all studies, with 2-steps in three [47, 48, 51] or 1 to 2-steps in the remainder [28, 49]. Amoxicillin only was used in two studies [26, 29], whilst the other three used other studies used other penicillin-class drugs [28, 49] and/or cephalexin [32, 47] (Table 2). A detailed description of direct DC and PST protocols is provided in Table 3.

**Table 2:** Interventions & baselines of participants in the included studies

**Study**	**Treatment arm**	**Comparator arm**	**Female sex**	**Age**	**Condition requiring penicillin**
Iammatteo et al. (2019) [25]	2-step direct DC to oral amoxicillin (*n* = 155)	PST followed by a challenge to amoxicillin (*n* = 142) or cephalexin (*n* = 37) (*n* = 170; some patients were tested with both amoxicillin and cephalexin)	DC: 77.4% (120) PST: 80% (136)	DC: 50.1 ± 2.4 PST: 52.1 ± 1.5	Not specified
Mustafa et al. (2019) [26]	2-step direct DC to oral amoxicillin (*n* = 79)	PST followed by challenge to amoxicillin (*n* = 80)	69.8% (111)	38.2 ± 25.0	Not specified
Stevenson et al. (2019) [27]	1 or 2-step direct DC to oral amoxicillin or culprit penicillin (*n* = 167)	PST followed by challenge to amoxicillin or culprit penicillin (*n* = 280)	DC: 55.7% (93) PST: 68.6% (192)	DC: 42.4 ± 19.5 PST: 47.0 ± 18.3	Not specified
Copaescu et al. (2023) (PALACE) [28]	1 or 2-step direct DC with oral amoxicillin, penicillin V, or flucloxacyllin (*n* = 188)	PST followed by 1-step oral challenge (*n* = 190)	65.5% (247)	51 (35-66)	Not specified
Ramsey et al. (2023) [29]	2-step direct DC with oral amoxicillin (*n* = 16)	PST followed by a challenge to amoxicillin (*n* = 22)	100% (38)	28.4 (25.2–30.8)	Peripartum prophylaxis in group B strept-colonized women

*DC* drug challenge, *PST* penicillin skin testing

**Table 3:** Detailed description of drug-challenge and skin testing controls

**Study**	**Drug-challenge**	**Skin test**	**Timing of outcome assessment**
Iammatteo et al. (2019) [25]	All graded challenges were single-blind (patient) and placebo-controlled Patients informed they would receive a placebo, but not told when it would be administered. All patients first received placebo followed by a 30-min observation period. Oral challenge was subsequently performed with 80 mg (1 mL of 400 mg/5 mL suspension) of amoxicillin followed by 30-min observation and subsequent administration of full therapeutic dose (500 mg) of amoxicillin. Patients were observed for 60 min after that. Amoxicillin was supplied in liquid formulation and mixed in the same yogurt and pharmaceutical sweetener used for placebo	PST were performed when appropriate before challenges according to published guidelines. Patients with PST positive for benzylpenicilloyl polylysine, penicillin G, piperacillin, or ampicillin but negative for cephalosporins underwent cephalexin graded challenges. In patients with positive cephalosporin skin testing but negative PST, graded challenges to amoxicillin were subsequently performed single-blind. All graded challenges were single-blind (patient) and placebo-controlled	DC: 120 min PST: not specified 61.9% (*n* = 96) patients were contacted within one month to assess for delayed reactions 44.5% (*n* = 69) patients were eligible for 1-year follow-up phone calls, and 19 were reached
Mustafa et al. (2019) [26]	Oral DC: 1/10 of the target dose of amoxicillin + monitoring for 30 min, and then full-dose amoxicillin + monitoring for 30 min Adults 40 and 400 mg; children 20 and 200 mg, or adult-dose depending on age, weight, and provider preference	PST: skin prick test to volar forearm using the Quintip testing device with benzylpenicilloyl polylysine as the major determinant, penicillin G 10,000 U/mL as the minor determinant, histamine 6 mg/mL as the positive control, and sodium chloride 0.9% as the negative control Negative skin prick testing was followed by intradermal testing administered on the upper arm with the same materials except a histamine concentration of 0.02 mg/mL	DC: 66.7 ± 4.8 min PST: 72.7 ± 5.3 min
Stevenson et al. (2019) [27]	Oral amoxicillin challenge was performed for patients with unspecified penicillin allergies. When the implicated penicillin was known, it was used. Challenge was performed with 1 or 2 doses (10% or full dose), with minimum 30-min observation between doses, and min 1 h after the final dose. Patients with history of anaphylaxis initially received 1% dose at some centers. Patients who tolerated the direct OPC were discharged on a course of penicillin (i.e., extended challenge) to assess for delayed reactions	PST was selected for each patient according to site-specific protocols, comprising benzylpenicillin (6 mg/mL), amoxicillin (20 mg/mL), and (where specified) the culprit penicillin(s); 5 sites also performed neat penicilloyl polylsysine and minor determinant mixture skin testing	DC: 60 to 90 min PST: not specified Phone follow-up 3 to 7 days after
Copaescu et al. (2023) (PALACE) [28]	Oral DC: lowest available therapeutic dose at each site of oral penicillin: Amoxicillin 250–500 mg in 85%, amoxicilin 400 mg 2-step in 6%, and penicillin VK 250–500 mg in 9%, and flucloxacillin 250 mg in 1% 60-min observation	PST: skin prick test with benzylpenicilloyl polylysine as the major determinant, ampicillin 25 mg/mL or Penicillin G 10,000 U/mL as minor determinants, histamine 10 mg/mL as positive control, and sodium chloride 0.9% as negative control. Test was read at 15 min Negative skin prick testing was followed by intradermal testing 0.02 mL with same materials 15 min later, and read after 15 min	DC: 1.8 h (IQR 1.3–3.7) PST: 2.3 (IQR 1.7–5.5) All patients contacted at 5 days for delayed adverse events
Ramsey et al. (2023) [29]	N.A	N.A	DC 70 min PST 72 min

##### Appraisal of Evidence

The three included randomized controlled trials [48, 50, 51] had one domain with high risk of bias and ≥ 2 domains with unclear risk. The two non-randomized controlled trials [47, 49] were rated as moderate quality (Table S.1. Supplementary Material).

The GRADE framework was applied to assess the certainty of evidence (Table S.2. Supplementary Material).

##### Safety of the Different Delabeling Strategies

**Fig. 3:**
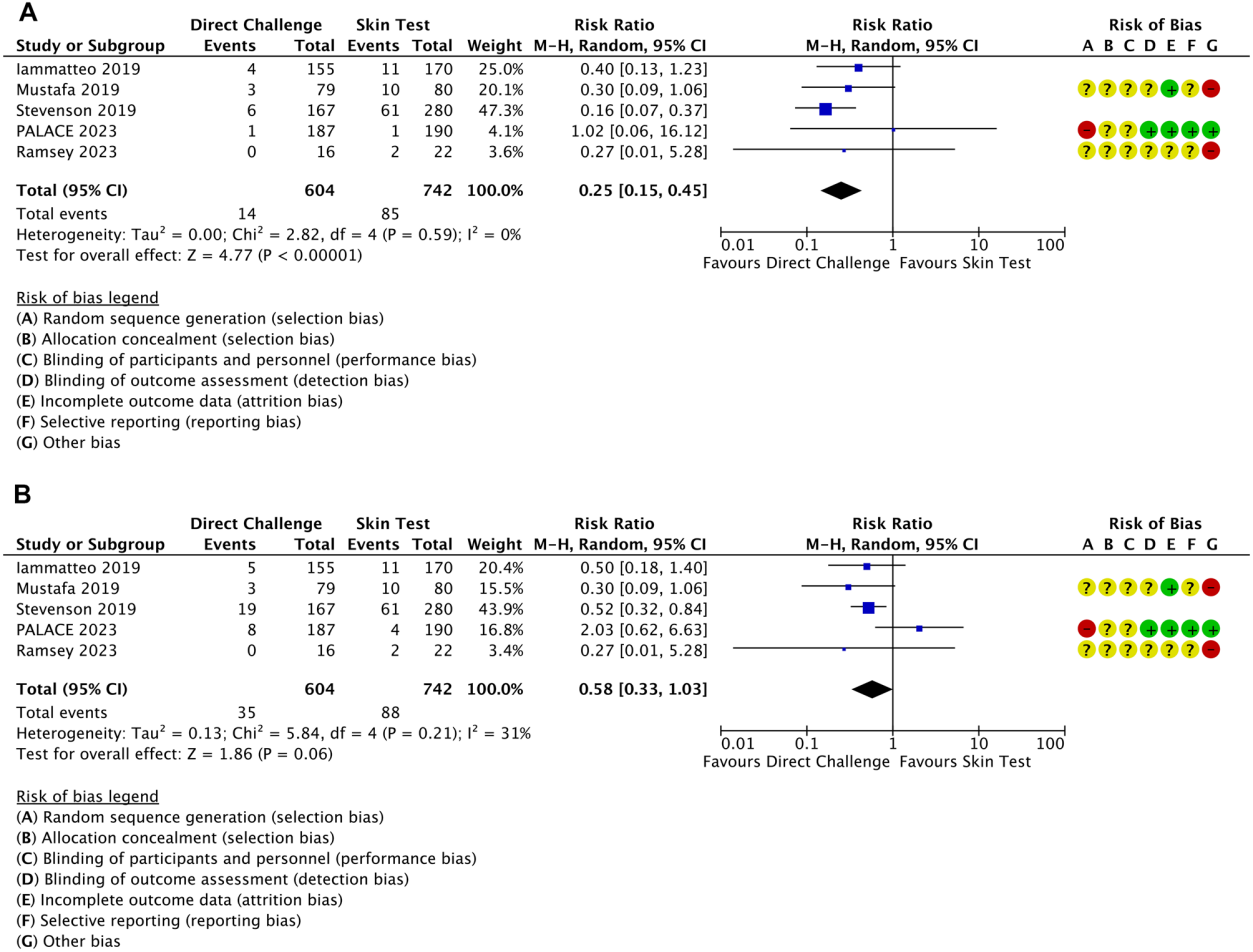
Forestplots with risk of bias assessment of the five studies (including three randomized controlled trials) for immediate allergic reactions (**A**) and delayed allergic reactions (**B**)

Immediate allergic reactions were observed for a minority of patients, usually of minor severity, and occurred less frequently in the DC group: 2.3% (14/604) vs. 11.5% (85/742); RR = 0.25, 95%CI 0.15–0.45, *P* < 0.00001 (Fig. 3A). Low heterogeneity was observed (*I*^2^ = 0%), and certainty of evidence was considered low due to double downgrading due to risk of bias.

No deaths or cases of anaphylaxis were reported. Despite not reporting any cases of anaphylaxis, one high-risk patient undergoing oral direct DC in Stevenson et al. developed a reaction classified as severe (grade 3 out of 4: lower respiratory not responding to inhaled corticoid, or upper respiratory with airway-associated edema with/without stridor) requiring adrenaline (49). Three female patients in the low-risk group undergoing direct DC required antihistamines to deal with test-related symptoms (coughing in one, and globus sensation in two). In this retrospective study, the rate of positive tests in the PST arm was much lower in the low-risk group (9/132, with 6 with positive skin test, and 3 patients with negative skin test but reacting to subsequent DC) than in the high-risk group (52/148, with 45 positive skin tests, and 7 patients with negative skin test reacting to the DC): 6.8% vs. 35.1%. This difference was not observed for DC, with 3.6% (4/112) in the low-risk, and 3.6% (2/55) in the high-risk group.

Iammatteo et al. reported four cases with allergic reaction to direct DC: three patients with mild rash and one patient with intractable pruritus that resolved within 1 h of antihistamines. In the historical control group, out of 11 patients with allergic reactions, 2 patients required antihistamines and 1 patient required corticosteroid to deal with angioedema, rash and/or erythema (47). Mustafa et al. (2019) reported three patients with reaction to direct DC consisting of cutaneous-only manifestations, all successfully treated with oral antihistamines (48). The ten positive PST were also cutaneous only reactions. No systemic reactions or need for adrenaline were observed in this study. The PALACE trial reported only one immediate reaction to testing in each group (50). Cutaneous events occurred in both groups during the first 5 days, with 9 patients in the direct DC group and 6 in the PST requiring anti-histamines, and 1 in the PST requiring intranasal corticosteroid. In Ramsey et al. (2023), two patients had positive PST, and no serious adverse events were described in any of the groups (51).

Three studies provided data on allergic reactions beyond the initial testing. Allergic reactions reaction on the longest available follow-up are numerically lower in the DC group than in the PST group: 5.8%, 35/604, vs. 12.5%, 88/742, OR = 0.58, 95%CI 0.33–1.03, *P* = 0.06 (Fig. 3B). Heterogeneity was moderate (*I*^2^ = 31%), and certainty of evidence was considered very low due to downgrading based on risk of bias, inconsistency, and imprecision. Iammatteo and colleagues reported that one of the patients with normal direct DC developed a rash after concurrently receiving penicillin and lidocaine for a dental procedure (47). Stevenson et al. described 207 patients with negative DC who underwent extended challenges. Of these, 6 of 144 in the low-risk group, and 6 of 63 in the high-risk group developed mild delayed cutaneous reactions (49). In the PALACE trial, during the 5-day follow-up, there were 22 adverse events in the direct DC group, with only 8 described as allergic (immediate diffuse rash/urticaria in 2 patients, and delayed diffuse rash/urticaria in 6 patients), and 24 adverse events in the PST group, with only 4 described as allergic (immediate diffuse rash urticaria in 1 patients, and delayed diffuse rash/urticaria in 3 patients) (50).

Non-allergic adverse events were reported by two trials. Iammatteo reported 14 patients with non-allergic reactions to direct DC: oropharingeal symptoms (*n* = 6), pruritus (*n* = 5), pruritus + oropharingeal symptoms (*n* = 1), sinus congestion (*n* = 1), nausea (*n* = 1), and mild pruritus + mild chest tightness (*n* = 1). In the historical control group, tingling sensation (*n* = 2), gastrointestinal symptoms (*n* = 1), and weakness/drowsiness/lightheadedness (*n* = 1) were among the reported non-allergic symptoms (47). In the PALACE trial, the reported non-allergic adverse events were antibiotic-associated non-immune reactions (6 in the DC group and 2 in the PST group), nausea/vomiting/diarrhea (2 in the direct DC group), and other unspecified nonsevere adverse events (6 in the direct DC group and 18 in the control group) (50).

No studies reported any vasovagal reactions, arrhythmias, or other severe adverse events. Data on antibiotic treatment adherence and acceptability to provider and patient were not provided in any study.

Two studies provided information on costs: Mustafa et al. estimated that each PST costed $393.66 whilst each DC costed $53.66 (48), nearly 8 times lower. Ramsey et al. Reported that cost for PST per patient was $301.73 whilst DC cost per patient was $187.46 (51).

Sensitivity and Subgroup-analyses for the endpoints immediate allergic reaction and allergic reaction at the longest available follow-up are presented in Table 4. Results show lower rate of allergic reactions from most scenarios with DC, except for studies in pregnancy and adult-only populations where DC and PST have similar rate of allergic reactions. The analysis of RCT only data shows a trend for lower rate of immediate allergic reactions, and comparable rates of delayed allergic reactions.

**Table 4:** Sensitivity and subgroup analyses

**Immediate allergic reaction**					
Sensitivity/subgroup analysis	%	RR	95%CI	*P*	*I*^2^
RCTs only (3 studies)	DC 4/282 PST 13/292	0.36	0.12–1.04	0.06	0%
Non-RCTs (2 studies)	DC 10/322 PST 72/450	0.24	0.10–0.56	0.001	37%
Pregnancy (1 study)	DC 0/16 PST 2/22	0.27	0.01–5.28	0.39	N.A
Children and adults (3 studies)	DC 13/401 PST 82/530	0.24	0.13–0.43	< 0.00001	0%
Adults only (2 studies)	DC 1/203 PST 3/212	0.55	0.07–4.16	0.56	0%
**Allergic reaction at longest available follow-up**					
Sensitivity/subgroup analysis	%	OR	95%CI	*P*	*I*^2^
RCTs only (3 studies)	DC 11/282 PST 16/292	0.66	0.15–2.91	0.58	61%
Non-RCTs (2 studies)	DC 24/322 PST 72/450	0.52	0.34–0.80	0.003	0%
Pregnancy (1 study)	DC 0/16 PST 2/22	0.27	0.01–5.28	0.39	N.A
Children and adults (3 studies)	DC 27/401 PST 82/530	0.49	0.32–0.74	0.0006	0%
Adults only (2 studies)	DC 8/203 PST 6/212	1.19	0.21–6.90	0.85	35%

## Discussion

We did not identify any studies assessing routine penicillin allergy testing for patients prescribed penicillin for secondary prevention of RHD. However, this topic has been previously covered by the WHO in its AWaRe antibiotic book: “Routine skin testing before prescribing a beta-lactam antibiotic (e.g. penicillin and amoxicillin) is not needed in children or adults and should not be recommended in guidelines as this is an unnecessary barrier to the use of Access antibiotics” [42].

Our systematic review showed that using PST or direct DC in low-risk individuals with history of penicillin allergy was safe as we did not observe any cases of anaphylaxis, death, or other severe adverse reactions with these approaches. Pooled data, suggested with low certainty, that direct oral DC may be a safe alternative to PST. A small percentage of patients required antihistamines, and only one was treated with adrenaline. These findings align with the WHO AWaRe antibiotic book recommendations: “direct oral challenge can be performed in carefully selected low-risk phenotypes” [42].

The evidence on the safety and effectiveness of penicillin allergy testing among the RHD population is missing; however, evidence from other populations can likely be generalized to RHD population. A systematic review reported a low prevalence of immediate reactions (1.98%) to penicillin derivatives among children reporting a β-lactam hypersensitivity [43]. Prevalence of any allergic reactions to may vary across countries and age groups, being higher in adults [43]. The population-weighted mean for a negative PST among inpatients with a penicillin allergy label was found to be 95.1% [31]. Skin tests and specific IgE quantification tests have high specificity, negative predictive value, and low sensitivity for confirming penicillin allergy [45]. Direct oral DC among people with a penicillin allergy label seems to be safe, with severe reactions observed only very rarely. Presence of trained healthcare professionals prevented any fatalities [32]. Training may cover areas as effective administration of IM BPG, recognizing and treating anaphylaxis.

Implementation of testing for patients with suspected penicillin allergy in high-prevalence areas of RHD with limited resources may deal with logistical and feasibility issues. Even though economic studies find penicillin allergy testing to be cost-saving in EU and US [55–57], cost-effectiveness, and affordability of such program in low- and middle-income setting is yet to be assessed. Logistics for direct oral DC without prior PST seem to be simpler, and two studies have shown a cost reduction with this strategy [48, 51]. Decision rules like the PEN-FAST have been previously suggested to identify low-risk patients not requiring PST by a specialist [58].

Limited evidence in the setting of secondary prophylaxis shows a very low incidence of anaphylaxis (1 to 3 cases per every 1000 individuals treated, and < 0.1% of all administered doses) in the setting of penicillin G benzathine administration [3]. A recent trial of BPG prophylaxis conducted between 2018 and 2020 among 458 Ugandan children and adolescents with latent RHD, only 1 participant (0.2% of participants in prophylaxis group and < 0.1% of the injections administered) showed symptoms of anaphylaxis (chest tightness and shortness of breath) 3 min after penicillin injection and the symptoms resolved after administering a single IM dose of epinephrine. Eight participants (1.7%) had a delayed hypersensitivity rash associated with penicillin, so their prophylactic antibiotic subsequently was changed to erythromycin [3]. This study implies that serious reactions are rare, immediate, and treatable.

A recent spatial modelling study showed that important variation in antibiotic usage between low-income and middle-income countries and high inequalities within some countries, with consumption of penicillins varying the highest between countries [59]. Consumption of broad-spectrum penicillin increased nearly three-fold in Sub-Saharan Africa between 2000 and 2018. Klein and colleagues showed that broad-spectrum penicillins were the most commonly consumed class of antibiotics (corresponding to 39% in 2015), and their use had increased 36% globally between 2000 and 2015 [60]. These data and high utilization explain why albeit rare, severe adverse reactions are bound to be observed in clinical practice and a reason for concern [23].

Sensitization, with loss of specific IgE and of positive PST and even tolerance to DC, can occur in many patients with a positive history of penicillin allergy after avoiding exposure to the drug [61]. Patients with a selective response to amoxicillin appear to lose sensitivity faster than those who responded to several penicillin determinants [62]. Adding further complexity to this matter, and to the topic of delabeling, these individuals can still, on rare occasions, redevelop a positive PST after subsequent exposure (i.e., resensitization) [63].

Analysis of Kaiser Permanente Southern California health plan members penicillin-class antibiotic use between 2009 and 2017 shows that among 6.1 million exposed to 5,617,402 courses of oral penicillins and 370,478 courses of parenteral penicillins, during a total of > 37 million patient-years of follow-up, there were 22 cases of anaphylaxis associated with oral penicillin exposure (1 in 255,320; 16 amoxicillin, 4 amoxicillin-clavulanate, and 2 dicloxacillin-associated) and 3 cases of anaphylaxis associated with parenteral penicillin exposure (1 in 123,792; 1 ampicillin-sulbactam and 2 piperacillin-tazobactam-associated) [64]. This analysis of patients from low-prevalence areas for RHD, but including 348,436 courses of oral penicillin and 93,466 courses of parenteral penicillin, suggests that albeit rare, anaphylaxis to penicillin-class antibiotics may be more common with parenteral exposure. Furthermore, new allergy reports within 30 days per course were low (0.61% with oral amoxicillin or ampicillin, 0.52% with oral penicillin, and 0.60% with parenteral penicillin).

A literature review and survey of experts with analysis of severe complications occurring following IM BPG suggests that some of these reactions may, in fact, be something other than anaphylaxis [20]. Suggested putative mechanisms for these reactions were the following: issues with preparation (powdered BPG, additives, diluents, or impurities), administration technique (e.g., accidental intravascular administration, poor technique causing too much pain), vasovagal-driven cardiac events or sudden death related to structural heart disease [20]. The 2022 American Heart Association Presidential Advisory reinforced that these reactions appear more frequent in patients with severe forms of RHD, and suggesting oral antibiotics for in such cases (e.g., severe mitral stenosis, severe aortic stenosis or insufficiency, ventricular dysfunction, and/or severe symptoms) and defining best practices for BPG administration [65].

Importance of the findings of this systematic review goes beyond RF and RHD, as BPG is also used for the treatment of Syphilis and other endemic Treponemal diseases in low-income countries. Furthermore, worldwide usage of amoxicillin is still high and its indications broad, including ear, nose, and throat infections [66, 67]; *Helicobacter pylori* eradication [68], lower respiratory [69], and urinary tract infections [70]; and skin and soft tissue infections [71], as well as prevention of endocarditis prior to dental procedures in high risk patients [72]. In consequence, penicillin allergy testing of selected patients will improve antimicrobial stewardship worldwide.

## Conclusion

Evidence on the safety and effectiveness of routine penicillin allergy testing among the RHD population is missing. However, severe allergic reactions to penicillin are extremely rare, and routine allergy testing has been discouraged in previous WHO guidance as it may create an unnecessary barrier to the use of antibiotics. Trained healthcare workers can effectively recognize and deal with severe allergic reactions.

Amoxicillin and benzathine penicillin G are still frequently used and have a broad range of indications for the prevention and treatment of infectious disease. Confirmation of penicillin allergy diagnosis or delabeling using PST or direct oral DC seems to be safe and is associated with a low rate of adverse reactions. 

### Supplementary Information

Below is the link to the electronic supplementary material.

Supplementary file1 (DOCX 306 KB)

## Data Availability

No datasets were generated or analysed during the current study.
