# Supplementary material for: Penicillin Allergy Testing and Delabeling for Patients Who Are Prescribed Penicillin: A Systematic Review for a World Health Organization Guideline
Source: Clin Rev Allergy Immunol. 2024 May 2;66(2):223–40. doi: 10.1007/s12016-024-08988-2 (PMC11193836; doi:10.1007/s12016-024-08988-2)
Supplement: Supplementary file 1 — Supplementary file1 (DOCX 306 KB) [file 12016_2024_8988_MOESM1_ESM.docx]

**Supplementary files**

**Table S.1.** Appraisal of Studies

|  | Immatteo et al. - Effective Public Health Practice Project Quality Assessment Tool  Rating: Moderate |
| --- | --- |
| Selection bias | Individuals are somewhat likely to be representative and all consecutive eligible non-high-risk patients were included. Rating: Moderate |
| Study design | Controlled clinical trial. Non-randomized. Rating: Moderate |
| Confounders | Minor differences were observed when comparing baselines of both treatment arms. Rating: Moderate |
| Blinding | The outcome assessor was aware of the research question and the study participants were aware of the research question. Rating: Weak |
| Data Collection Methods | Outcome data was objectively collected by the investigators. No information on interobserver variability. Rating: Moderate |
| Withdrawals and Drop-outs | Outcome data was available for immediate/short-term allergy. However, longterm data was missing for most patients. Rating: Weak |
| Intervention Integrity | Patients received the allocated intervention. Rating: Strong |
| Analyses | Quantitative analysis was appropriate to the research question, but data on historical controls should have also been presented on the paper. Data on patients receiving cephalosporins should have also been presented in separate. Rating: Weak |
|  | Stevenson et al. - Effective Public Health Practice Project Quality Assessment Tool  Rating: Moderate |
| Selection bias | Individuals are likely to be very representative and all consecutive eligible patients were included. Rating: Strong |
| Study design | Case-control study. Rating: Moderate |
| Confounders | Minor differences were observed when comparing baselines of both treatment arms. Rating: Moderate |
| Blinding | The outcome assessor was aware of the research question. Study participants were not aware of the research question as this was a restrospective study. Rating: Weak |
| Data Collection Methods | Outcome data was objectively collected by the investigators. No information on interobserver variability. Rating: Moderate |
| Withdrawals and Drop-outs | Outcome data was available for immediate/short-term allergy. Longterm data was available for most patients in the extended challenge group. Rating: Moderate |
| Intervention Integrity | Patients received the allocated intervention. Rating: Strong |
| Analyses | Quantitative analysis was appropriate to the research question. Data presentation on adverse events is not entirely clear at times. Rating: Moderate |
|  | Mustafa et al. Risk of Bias Tool |
| Random sequence generation | No information provided. Unclear risk of selection bias |
| Allocation concealment | No information provided. Unclear risk of selection bias. |
| Blinding of participants and personnel | No information provided. Unclear risk of performance bias. |
| Blinding of outcome assessment | No information provided. Unclear risk of detection bias. |
| Incomplete outcome data | All endpoints were assessed on the same visit.Low risk of attrition bias |
| Selective reporting | No information provided / no protocol online with pre-planned outcomes. Unclear risk of reporting bias |
| Other bias | No registration in clinicaltrials.gov or other trial database. High risk for Other Bias. |
|  | PALACE – Risk of Bias Tool |
| Random sequence generation | *"Randomisation will be non-blinded and performed using REDCap (Research Electronic Data Capture)."* Use of permuted blocks increases the probability of correctly guessing the next allocation compared to other methods. High risk of Selection bias. |
| Allocation concealment | The allocation sequence will be developed and uploaded to REDCap by a trial statistician and will remain concealed." Method of concealment not specified. Unclear risk of Selection bias |
| Blinding of participants and personnel | Study participants/personnel are not blinded. Uncertain if this can lead to any bias. Unclear risk of Performance bias |
| Blinding of outcome assessment | Outcome assessors were unaware of study group allocations. Low risk of detection bias |
| Incomplete outcome data | All outcomes assessed immediately or at 5 days. Low risk of attrition bias. |
| Selective reporting | Published outcomes were all reported in the published protocol and clinicaltrials.gov. Low risk of reporting bias. |
| Other bias | Published protocol and trial registered on clinicaltrials.gov - NCT04454229. Low risk |
|  | Ramsey et al. Risk of Bias Tool |
| Random sequence generation | No information provided. Unclear risk of selection bias |
| Allocation concealment | No information provided. Unclear risk of selection bias. |
| Blinding of participants and personnel | No information provided. Unclear risk of performance bias. |
| Blinding of outcome assessment | No information provided. Unclear risk of detection bias. |
| Incomplete outcome data | No information provided. Unclear risk of attrition bias |
| Selective reporting | No information provided / no protocol online with pre-planned outcomes. Reported outcomes are similar to study by the same group in 2019. Unclear risk of reporting bias |
| Other bias | No registration in clinicaltrials.gov or other trial database. High risk for Other Bias. |

**Table S.2.** GRADE Summary of Findings Tables


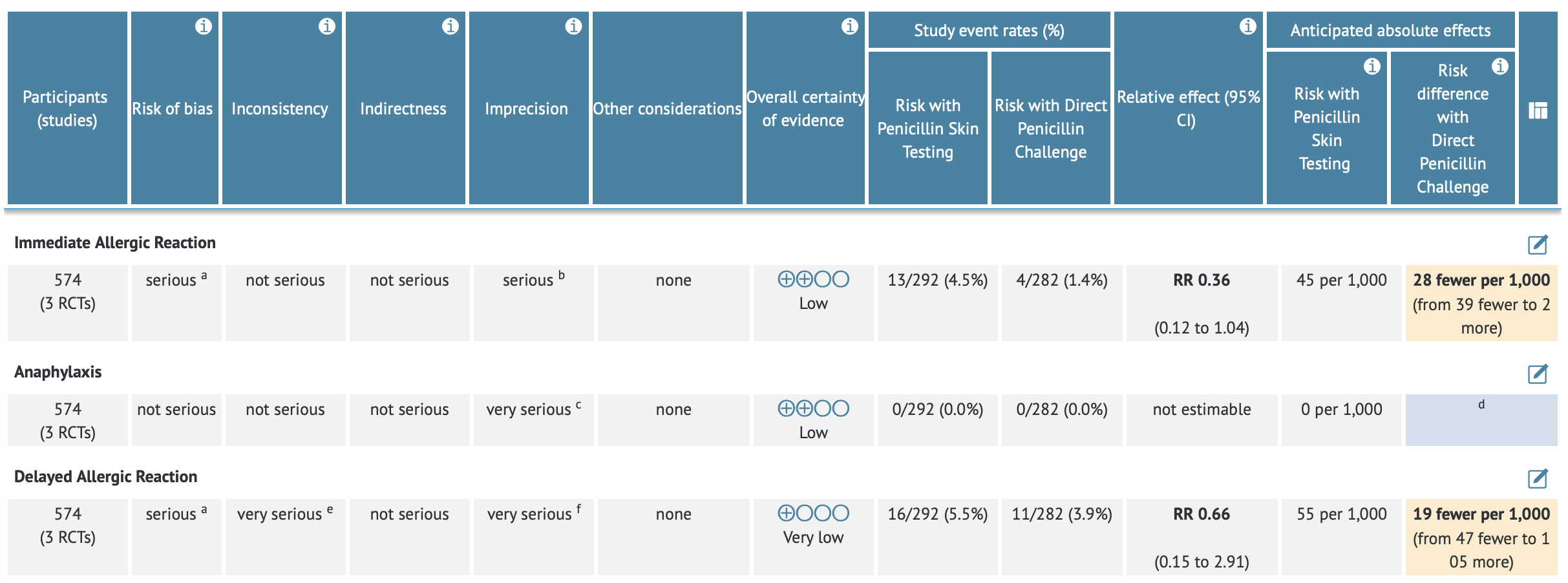


**Notes:**

1. All trials had multiple domains (≥2) with unclear risk, and one high risk domain. Downgrade 1 level for potentially subjective outcomes.
2. Broad 95%CI confidence interval: 0.12 to 1.04
3. Anaphylaxis in this setting is quite rare and, with no observed events, the 95% confidence interval could not be estimated.
4. No events in any treatment arm
5. High heterogeneity - I^2^ 61%.
6. Very Broad 95% confidence interval: 0.15 to 2.91.

***Appendix 1: Search Strategies***

**CENTRAL**

Date Run: 02/10/2022 18:20:39

#1 ([mh "Rheumatic Heart Disease"] OR [mh "Rheumatic Fever"] OR (Rheumatic Card* OR Rheumatic Fever* OR Rheumatic Heart OR Rheumatoid Fever* OR Rheumatic Valv* OR Rheumatic Pancarditis OR Rheumatic Endocarditis OR Rheumatic Myocarditis OR Rheumatic Pericarditis OR Rheumatoid Pancarditis OR Rheumatoid Endocarditis OR Rheumatoid Myocarditis OR Rheumatoid Pericarditis OR Rheumatoid Card* OR Rheumatoid Heart OR Rheumatoid Valv*):ti,ab,kw) AND ([mh Hypersensitivity] or [mh "Drug Hypersensitivity"] or [mh "Hypersensitivity, Delayed"] or [mh "Hypersensitivity, Immediate"] or [mh "Skin Tests"] or [mh "Patch Tests"] or [mh "Intradermal Tests"] or [mh "Basophil Degranulation Test"] or [mh "Flow Cytometry"] or [mh "Enzyme-Linked Immunosorbent Assay"] or [mh Radioimmunoassay] or [mh "Immunoenzyme Techniques"] or [mh "Radioallergosorbent Test"] or [mh Tryptases] or (Hypersensitiv* or Allergy or Allergies or Allergic or Epicutaneous Test* or Epidermal Test* or Intradermal Test* or Patch Test* or Percutaneous Test* or Skin Prick Test* or Graded Challenge or Test Dosing or Tryptase* or Trypsin Like Activit* or Trypsin Like Enzyme* or Trypsin Like Protease* or Trypsin Like Proteinase* or "E.C. 3.4.21.59" or Hypererg* or Hypersensibilit* or Immediate Type Reaction* or Delayed Sensitivity Reaction* or Delayed Sensitivity Type* or Delayed Type Reaction* or Drug Intolerance* or Intracutaneous Reaction* or Intracutaneous Test* or Intradermal Test* or Passive Transfer Test* or Prausnitz Kustner Test* or Antigenicit* or Basophil Activating Test* or Basophil Activation Assay* or Basophil Activation Test* or Basophil Activity Test* or Basophile Activation Assay* or Basophile Activation Test* or Basophiles Activation Test* or Basophilic Activation Test* or Basophils Activation Assay* or Basophils Activation Test* or Cellular Antigen Stimulation Test* or Histamine Release Test* or Leucocyte Transformation Test* or Leukocyte Transformation Test* or Lymphoblast Stimulation Test* or Lymphoblastic Transformation Test* or Lymphocytal Transformation Test* or Lymphocyte Stimulation Test* or Lymphocyte Transformation Test* or Lymphocytic Transformation Test* or Flow Cytometr* or Flow Microfluorometr* or Flow Microfluorimetr* or Flow Cytofluorometr* or Fluorescence Activated Cell Sorting* or ELISA or ELISpot or Enzyme Labeled Immunosorbent Assay* or Enzyme-Linked Immunosorbent Spot Assay* or Enzyme Linked Immune Assay* or Enzyme Linked Immuno Assay* or Enzyme Linked Immunoassay* or Enzyme Linked Immunosorbent Assay* or Enzyme Linked Immunospecific Assay* or Provocation Test* or Challenge Test* or Stimulation Test* or FEIA or Enzyme Immunoassay* or EIA or Immunoenzyme Method* or Immunoenzyme Technique* or Radioimmunoassay* or Radioallergosorbent Test* or Radio Allergo Sorbent Technique* or Radioallergosorbent Technique* or RAST or Radioimmune Assay* or Radioimmunochemical Assay* or Radioimmunodetection or Radioimmunosorbent Test* or RIA or RIST or Radioimmunosorbent Assay* or Allergens Radioimmunosorbent Assay*):ti,ab,kw) in Trials 251

**ClinicalTrials.gov**

Advanced Search

Condition or disease: Rheumatic Fever OR Rheumatic Cardiac OR Rheumatic Heart OR Rheumatoid Fever OR Rheumatic Valve OR Rheumatic Myocarditis OR Rheumatoid Heart OR Rheumatoid Valve

Study type: Interventional Studies (Clinical Trials)

78 Studies found

**Database: Embase <1974 to 2022 September 30>**

1 Rheumatic Fever/ or exp Rheumatic Heart Disease/ or exp Rheumatic Carditis/ or Rheumatic Endocarditis/ or Rheumatic Myocarditis/ or Rheumatic Pericarditis/ or Rheumatoid Nodule/ or (Rheumatic Card* or Rheumatic Fever* or Rheumatic Heart or Rheumatoid Fever* or Rheumatic Valv* or Rheumatic Pancarditis or Rheumatic Endocarditis or Rheumatic Myocarditis or Rheumatic Pericarditis or Rheumatoid Pancarditis or Rheumatoid Endocarditis or Rheumatoid Myocarditis or Rheumatoid Pericarditis or Rheumatoid Card* or Rheumatoid Heart or Rheumatoid Valv*).mp. (22754)

2 Hypersensitivity/ or Skin Test/ or Patch Test/ or Immediate Type Hypersensitivity/ or Allergic Reaction/ or Delayed Hypersensitivity/ or Drug Hypersensitivity/ or Penicillin Allergy/ or Intracutaneous Test/ or Basophil Activation Test/ or exp Allergy Test/ or Allergy ELISA/ or Lymphocyte Transformation Test/ or exp Flow Cytometry/ or Enzyme Linked Immunosorbent Assay/ or Provocation Test/ or Allergy Rapid Test/ or Allergy Test Kit/ or Allergy ELISA Kit/ or Enzyme Immunoassay/ or Radioimmunoassay/ or Radioallergosorbent Test/ or Radioimmunoassay Kit/ or Tryptase/ or Tryptase Test Kit/ or (Hypersensitiv* or Allergy or Allergies or Allergic or Epicutaneous Test* or Epidermal Test* or Intradermal Test* or Patch Test* or Percutaneous Test* or Skin Prick Test* or Graded Challenge or Test Dosing or Tryptase* or Trypsin Like Activit* or Trypsin Like Enzyme* or Trypsin Like Protease* or Trypsin Like Proteinase* or "E.C. 3.4.21.59" or Hypererg* or Hypersensibilit* or Immediate Type Reaction* or Delayed Sensitivity Reaction* or Delayed Sensitivity Type* or Delayed Type Reaction* or Drug Intolerance* or Intracutaneous Reaction* or Intracutaneous Test* or Intradermal Test* or Passive Transfer Test* or Prausnitz Kustner Test* or Antigenicit* or Basophil Activating Test* or Basophil Activation Assay* or Basophil Activation Test* or Basophil Activity Test* or Basophile Activation Assay* or Basophile Activation Test* or Basophiles Activation Test* or Basophilic Activation Test* or Basophils Activation Assay* or Basophils Activation Test* or Cellular Antigen Stimulation Test* or Histamine Release Test* or Leucocyte Transformation Test* or Leukocyte Transformation Test* or Lymphoblast Stimulation Test* or Lymphoblastic Transformation Test* or Lymphocytal Transformation Test* or Lymphocyte Stimulation Test* or Lymphocyte Transformation Test* or Lymphocytic Transformation Test* or Flow Cytometr* or Flow Microfluorometr* or Flow Microfluorimetr* or Flow Cytofluorometr* or Fluorescence Activated Cell Sorting* or ELISA or ELISpot or Enzyme Labeled Immunosorbent Assay* or Enzyme-Linked Immunosorbent Spot Assay* or Enzyme Linked Immune Assay* or Enzyme Linked Immuno Assay* or Enzyme Linked Immunoassay* or Enzyme Linked Immunosorbent Assay* or Enzyme Linked Immunospecific Assay* or Provocation Test* or Challenge Test* or Stimulation Test* or FEIA or Enzyme Immunoassay* or EIA or Immunoenzyme Method* or Immunoenzyme Technique* or Radioimmunoassay* or Radioallergosorbent Test* or Radio Allergo Sorbent Technique* or Radioallergosorbent Technique* or RAST or Radioimmune Assay* or Radioimmunochemical Assay* or Radioimmunodetection or Radioimmunosorbent Test* or RIA or RIST or Radioimmunosorbent Assay* or Allergens Radioimmunosorbent Assay*).mp. (1633574)

3 1 and 2 (1157)

4 (rat or rats or mouse or mice or swine or porcine or murine or sheep or lambs or pigs or piglets or rabbit or rabbits or cat or cats or dog or dogs or cattle or bovine or monkey or monkeys or trout or marmoset$1).ti. and animal experiment/ (1167889)

5 Animal experiment/ not (human experiment/ or human/) (2451550)

6 4 or 5 (2513280)

7 3 not 6 (1134)

**ISRCTN**

Advanced Search

No results found for "Rheumatic Fever AND ( Condition: Rheumatic Fever )"

No results found for "( Condition: Rheumatic Cardiac )"

3 results within Condition: Rheumatic Heart

No results found for "( Condition: Rheumatoid Fever )"

No results found for "( Condition: Rheumatic Valve )"

No results found for "( Condition: Rheumatic Myocarditis )"

No results found for "( Condition: Rheumatoid Heart )"

No results found for "( Condition: Rheumatoid Valve )"

**Database: Ovid MEDLINE(R) ALL <1946 to September 30, 2022>**

1 Rheumatic Heart Disease/ or exp Rheumatic Fever/ or (Rheumatic Card* or Rheumatic Fever* or Rheumatic Heart or Rheumatoid Fever* or Rheumatic Valv* or Rheumatic Pancarditis or Rheumatic Endocarditis or Rheumatic Myocarditis or Rheumatic Pericarditis or Rheumatoid Pancarditis or Rheumatoid Endocarditis or Rheumatoid Myocarditis or Rheumatoid Pericarditis or Rheumatoid Card* or Rheumatoid Heart or Rheumatoid Valv*).mp. (26046)

2 Hypersensitivity/ or Drug Hypersensitivity/ or Hypersensitivity, Delayed/ or Hypersensitivity, Immediate/ or Skin Tests/ or Patch Tests/ or Intradermal Tests/ or Basophil Degranulation Test/ or Flow Cytometry/ or exp Enzyme-Linked Immunosorbent Assay/ or exp Radioimmunoassay/ or Immunoenzyme Techniques/ or Radioallergosorbent Test/ or Tryptases/ or (Hypersensitiv* or Allergy or Allergies or Allergic or Epicutaneous Test* or Epidermal Test* or Intradermal Test* or Patch Test* or Percutaneous Test* or Skin Prick Test* or Graded Challenge or Test Dosing or Tryptase* or Trypsin Like Activit* or Trypsin Like Enzyme* or Trypsin Like Protease* or Trypsin Like Proteinase* or "E.C. 3.4.21.59" or Hypererg* or Hypersensibilit* or Immediate Type Reaction* or Delayed Sensitivity Reaction* or Delayed Sensitivity Type* or Delayed Type Reaction* or Drug Intolerance* or Intracutaneous Reaction* or Intracutaneous Test* or Intradermal Test* or Passive Transfer Test* or Prausnitz Kustner Test* or Antigenicit* or Basophil Activating Test* or Basophil Activation Assay* or Basophil Activation Test* or Basophil Activity Test* or Basophile Activation Assay* or Basophile Activation Test* or Basophiles Activation Test* or Basophilic Activation Test* or Basophils Activation Assay* or Basophils Activation Test* or Cellular Antigen Stimulation Test* or Histamine Release Test* or Leucocyte Transformation Test* or Leukocyte Transformation Test* or Lymphoblast Stimulation Test* or Lymphoblastic Transformation Test* or Lymphocytal Transformation Test* or Lymphocyte Stimulation Test* or Lymphocyte Transformation Test* or Lymphocytic Transformation Test* or Flow Cytometr* or Flow Microfluorometr* or Flow Microfluorimetr* or Flow Cytofluorometr* or Fluorescence Activated Cell Sorting* or ELISA or ELISpot or Enzyme Labeled Immunosorbent Assay* or Enzyme-Linked Immunosorbent Spot Assay* or Enzyme Linked Immune Assay* or Enzyme Linked Immuno Assay* or Enzyme Linked Immunoassay* or Enzyme Linked Immunosorbent Assay* or Enzyme Linked Immunospecific Assay* or Provocation Test* or Challenge Test* or Stimulation Test* or FEIA or Enzyme Immunoassay* or EIA or Immunoenzyme Method* or Immunoenzyme Technique* or Radioimmunoassay* or Radioallergosorbent Test* or Radio Allergo Sorbent Technique* or Radioallergosorbent Technique* or RAST or Radioimmune Assay* or Radioimmunochemical Assay* or Radioimmunodetection or Radioimmunosorbent Test* or RIA or RIST or Radioimmunosorbent Assay* or Allergens Radioimmunosorbent Assay*).mp. (1087444)

3 1 and 2 (790)

4 exp Animals/ not Humans.sh. (5052290)

5 3 not 4 (758)

**WHO ICTRP**

Advanced Search

Rheumatic Fever OR Rheumatic Cardiac OR Rheumatic Heart OR Rheumatoid Fever OR Rheumatic Valve OR Rheumatic Myocarditis OR Rheumatoid Heart OR Rheumatoid Valve in the Condition

Recruitment status is ALL

144 records for 142 trials found

**Conference Proceedings Citation Index-Science=CPCI-S (1990 – present)**

(Rheumatic Card* or Rheumatic Fever* or Rheumatic Heart or Rheumatoid Fever* or Rheumatic Valv* or Rheumatic Pancarditis or Rheumatic Endocarditis or Rheumatic Myocarditis or Rheumatic Pericarditis or Rheumatoid Pancarditis or Rheumatoid Endocarditis or Rheumatoid Myocarditis or Rheumatoid Pericarditis or Rheumatoid Card* or Rheumatoid Heart or Rheumatoid Valv*) AND (Hypersensitiv* or Allergy or Allergies or Allergic or Epicutaneous Test* or Epidermal Test* or Intradermal Test* or Patch Test* or Percutaneous Test* or Skin Prick Test* or Graded Challenge or Test Dosing or Tryptase* or Trypsin Like Activit* or Trypsin Like Enzyme* or Trypsin Like Protease* or Trypsin Like Proteinase* or "E.C. 3.4.21.59" or Hypererg* or Hypersensibilit* or Immediate Type Reaction* or Delayed Sensitivity Reaction* or Delayed Sensitivity Type* or Delayed Type Reaction* or Drug Intolerance* or Intracutaneous Reaction* or Intracutaneous Test* or Intradermal Test* or Passive Transfer Test* or Prausnitz Kustner Test* or Antigenicit* or Basophil Activating Test* or Basophil Activation Assay* or Basophil Activation Test* or Basophil Activity Test* or Basophile Activation Assay* or Basophile Activation Test* or Basophiles Activation Test* or Basophilic Activation Test* or Basophils Activation Assay* or Basophils Activation Test* or Cellular Antigen Stimulation Test* or Histamine Release Test* or Leucocyte Transformation Test* or Leukocyte Transformation Test* or Lymphoblast Stimulation Test* or Lymphoblastic Transformation Test* or Lymphocytal Transformation Test* or Lymphocyte Stimulation Test* or Lymphocyte Transformation Test* or Lymphocytic Transformation Test* or Flow Cytometr* or Flow Microfluorometr* or Flow Microfluorimetr* or Flow Cytofluorometr* or Fluorescence Activated Cell Sorting* or ELISA or ELISpot or Enzyme Labeled Immunosorbent Assay* or Enzyme-Linked Immunosorbent Spot Assay* or Enzyme Linked Immune Assay* or Enzyme Linked Immuno Assay* or Enzyme Linked Immunoassay* or Enzyme Linked Immunosorbent Assay* or Enzyme Linked Immunospecific Assay* or Provocation Test* or Challenge Test* or Stimulation Test* or FEIA or Enzyme Immunoassay* or EIA or Immunoenzyme Method* or Immunoenzyme Technique* or Radioimmunoassay* or Radioallergosorbent Test* or Radio Allergo Sorbent Technique* or Radioallergosorbent Technique* or RAST or Radioimmune Assay* or Radioimmunochemical Assay* or Radioimmunodetection or Radioimmunosorbent Test* or RIA or RIST or Radioimmunosorbent Assay* or Allergens Radioimmunosorbent Assay*) (Topic) 53

***Appendix 2: List of Excluded Studies***

**I. Routine allergy testing in patients with rheumatic heart disease and prescribed with Benzathine Penicillin G Injections**

Felix MMR, Kuschnir FC, Cunha AJLA. Sensitization to penicillin in adolescents with rheumatic fever. Journal Allergy Clinical Immunol Pract. 2011;1:AB194.

Felix M, Kuschnir F, Aoun N, Sztajnbok F, Cunha A. Evaluation of penicillin G sensitization in adolescents with rheumatic fever. Revista Portuguesa de Imunoalergologia. 2012;20:281-90.

Kaya A, Erkocoglu M, Senkon OG, et al. Confirmed penicillin allergy among patients receiving benzathine penicillin prophylaxis for acute rheumatic fever. Allergologia et Immunopathologia. 2014;42(4):289-92.

Markowitz M, Lue HC. Allergic reactions in rheumatic fever patients on long-term benzathine penicillin G: the role of skin testing for penicillin allergy. Pediatrics. 1996;97(6):981-3.

Regmi PR, Upadhyaya AB. Allergic reaction to long-term benzathine penicillin injection for secondary prevention of acute rheumatic fever and recommendations for skin testing. Circulation. 2012;125:e766.

Sanyahumbi A, Ali S, Benjamin IJ, et al. Penicillin Reactions in Patients With Severe Rheumatic Heart Disease: A Presidential Advisory From the American Heart Association. Journal of the American Heart Association. 2022;11(5):e024517.

**II. Penicillin allergy delabeling**

Chow TG, Patel G, Mohammed M, Johnson D, Khan DA. Delabeling penicillin allergy in a pediatric primary care clinic. Ann Allergy Asthma Immunol. 2023;130:667-9.

Li J, Cvetanovski V, Fernando S. Single-step direct drug provocation testing is safe for delabelling selected non-low-risk penicillin allergy labels. Ann Allergy Asthma Immunol.2021;127:232-5.

Petersen BT, Gradman J. Prospective study of 5-day challenge with penicillins in children. BMJ Paediatr Open. 2020;4(1):e000734.

Labrosse R, Paradis L, Samaan K, et al. Sensitivity and specificity of double-blinded penicillin skin testing in relation to oral provocation with amoxicillin in children. Allergy Asthma Clin Immunol. 2020;16(1):57.

Moreno-Borque R, Gonzalez MDMR, Lorduy TC. The Importance Of The Clinical History and Oral Challenge Against Misleading False-positive Supplementary Tests In The Study Of Drug Allergies. Journal Allergy Clinical Immunol Pract. 2020;8(Suppl):AB188.

Solenski R, Jacobs J, Lester M, et al. Penicillin Allergy Evaluation: A Prospective, Multicenter, Open-Label Evaluation of a Comprehensive Penicillin Skin Test Kit. . Journal Allergy Clinical Immunol Pract. 2019:7:1876-1885.e3.

Savic L, Gurr L, Kaura V, et al. Penicillin allergy de-labelling ahead of elective surgery: feasibility and barriers. Br J Anaesth. 2019;123:e110-e116.

Iammatteo M, Alvarez-Arango S, Ferastraoaru DE, et al. Oral graded challenges to amoxicillin without prior skin testing. Journal Allergy Clinical Immunol Pract. 2018;6(Suppl):AB34.

Labrosse R, Paradis LP, Samaan K, et al. Amoxicillin reuse after a five-day challenge for the evaluation of delayed-type penicillin allergy in children. Journal Allergy Clinical Immunol Pract. 2017;5(Suppl):AB32.

Mac YE, Goldberg B, Poon KYT. Use of commercial anti-penicillin IgE fluorometric enzyme immunoassays to diagnose penicillin allergy. Ann Allergy Asthma Immunol. 2010;105:136-141.

Goldberg A, Confino-Cohen R. Skin testing and oral penicillin challenge in patients with a history of remote penicillin allergy. Ann Allergy Asthma Immunol. 2008;100:37-43.

Torres MJ, Mayorga C, Leyva L, et al. Controlled administration of penicillin to patients with a positive history but negative skin and specific serum IgE tests. Clin Exp Allergy. 2002;32:270-276.

Torres J, Romano A, Mayorga C, et al. Diagnostic evaluation of a large group of patients with immediate allergy to penicillins: The role of skin testing. Allergy. 2001;56:850-6.

Cooper L, Harbour J, Sneddon J, Seaton RA. Safety and efficacy of de-labelling penicillin allergy in adults using direct oral challenge: A systematic review. JAC Antimicrob Resist. 2021;3:dlaa123.

DesBiens M, Scalia P, Ravikumar S, et al. A Closer Look at Penicillin Allergy History: Systematic Review and Meta-Analysis of Tolerance to Drug Challenge. Am J Med. 2020;133:452-462.e4.

Reilly CA, Backer G, Basta D, Riblet NBV, Hofley PM, Gallagher MC. The effect of preoperative penicillin allergy testing on perioperative non-beta-lactam antibiotic use: A systematic review and meta-analysis. Allergy Asthma Proc. 2018;39:420-429.

DesBiens M, Scalia P, Ravikumar S, Glick A, Erinne O. Most patients tolerate penicillin administration despite history of nonanaphylactic penicillin allergy: A systematic review and meta-analysis. Open Forum Infectious Diseases. 2018;5(Supplement 1):S554.

Hassoun-Kheir N, Bergman R, Weltfriend S. The use of patch tests in the diagnosis of delayed hypersensitivity drug eruptions. Int J Dermatol. 2016;55:1219-1224.

Jones BM, Hamlin A, Crosby J, Bland C. Clinical and economic outcome evaluation with penicillin skin testing as an antimicrobial stewardship initiative in a not-for-proft community health system. Open Forum Infectious Diseases. 2018;5(Supplement 1):S507.

Macy EM, Chen LH. The Incidence of Anaphylaxis Associated with Oral and Parenteral Penicillin-Class Antibiotic Exposures. Journal Allergy Clinical Immunol Pract. 2017;5(Suppl):AB33.

Sousa-Pinto B, Fernandes A, Araujo L, Fonseca JA, Freitas A, Delgado L. Clinical and economic burden of hospitalizations with registry of penicillin allergy. Journal Allergy Clinical Immunol Pract. 2017;5(Suppl):AB59.
